# Advanced Congestive Heart Failure Associated With Disseminated Intravascular Coagulopathy

**DOI:** 10.1177/2324709615623298

**Published:** 2015-12-29

**Authors:** Annahita Sarcon, Xiaoli Liu, David Ton, James Haywood, Todd Hitchcock

**Affiliations:** 1 Scripps Green Hospital, La Jolla, CA, USA

**Keywords:** disseminated intravascular coagulopathy, congestive heart failure

## Abstract

*Background*. Disseminated intravascular coagulopathy (DIC) is a complication of an underlying disease and not a primary illness. It is most commonly associated with sepsis, trauma, obstetrical complications, and malignancies. There are very few cases in the literature illustrating the association between DIC and congestive heart failure. *Findings*. In this report, we present a case of severe congestive heart failure, leading to biventricular thrombi and subsequently DIC. *Conclusion*. We suggest that the association between congestive heart failure and DIC is an underrecognized one. Congestive heart failure continues to remain a major cause of morbidity and mortality despite advances in medical therapies. Thus far, the precise role of coagulation factors in congestive heart failure is unknown. Further investigations are needed to elucidate the pathophysiology of congestive heart failure and coagulation factors.

## Introduction

Disseminated intravascular coagulopathy (DIC) is not a primary illness but a complication of an underlying disease. It is commonly associated with sepsis, trauma, malignancy, and obstetrical complications. In this report, we present a unique case of DIC in the setting of advanced congestive heart failure (CHF) with biventricular thrombi.

## Case Presentation

A 76-year-old woman with past medical history of transient ischemic attacks managed on Aggrenox presented with 2-week history of malaise and diffuse abdominal pain. Her physical exam on admission was significant for increased jugular venous pressure of 10 cm H_2_O, hepatojugular reflux, scant crackles at the lung bases, and 1+ pitting edema of the lower extremities.

**Figure 1.**
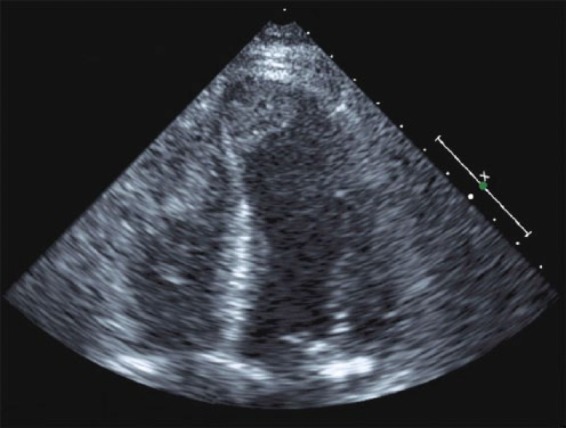
Two-dimensional transthoracic echocardiogram demonstrating large biventricular mural thrombi.

**Figure 2.**
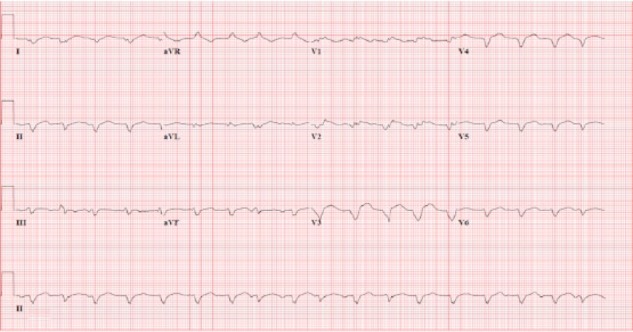
EKG revealing Q-waves in V1-V6 suggestive of a prior anterior myocardial infarction.

Laboratory data showed mild leukocytosis and moderately elevated liver enzymes and creatinine, which were normal 2 months prior. Lipase and the hepatitis viral panel were unrevealing. A noncontrast computed tomography of abdomen and pelvis showed nonspecific colitis. An abdominal ultrasound revealed moderate hepatomegaly, and sluggish flow through the portal venous system without biliary dilatation. Empiric antibiotics were initiated for presumed acute gastroenteritis and infectious colitis. However, the patient continued to decline despite supportive medical therapy. A magnetic resonance imaging scan of the abdomen was obtained, which showed hepatomegaly, but a cardiac view revealed a ventricular filling defect and biventricular mural thrombi. This was confirmed with a transthoracic echocardiogram (EKG) demonstrating large biventricular mural thrombi, and apical akinesis with an estimated ejection fraction (EF) of 10% (Figure 1); previous EF was 55% 2 years prior. The EKG revealed Q-waves in V1-V6, suggestive of a prior anterior myocardial infarction (Figure 2). Further laboratory data revealed DIC. However, the remainder diagnostic studies were unrevealing, including infections, hypercoagulable state, and malignancy. Over the course of hospitalization, the patient’s liver and renal function continued to decline despite supportive medical therapy. Given the overall poor prognosis and patient’s wishes, comfort measures were enacted and the patient expired 1 week later.

## Conclusion

While CHF and DIC are common clinical entities, the association between the two is not readily recognized. The first report by Heckman and Rosove, in 1980, described the association between massive ventricular thrombi and DIC in CHF.^1^ Years later, Solomon et al^2^ published a case series demonstrating DIC associated with large ventricular thrombi after myocardial infarction. Nonetheless, this critical association remains underrecognized and only few recent reports exist in the literature. A recent case report and literature review in 2015 suggests that in patients presenting DIC, severe biventricular nonischemic cardiac dysfunction with intracardiac thrombi should indeed be considered.^3^ Presumably activation of the neuroendocrine system in CHF is responsible for coagulation pathway activation, endothelial dysfunction, and increased pro-inflammatory cytokine levels.^4-6^ Other studies have also observed increased coagulation factors, fibrinolysis, and platelet activation.^7^ The ultimate underlying mechanism may be due to tissue ischemia, stagnant blood flow, and increased α-adrenergic activity in CHF leading to DIC. Ventricular thrombi can be a consequence of activated coagulation pathway but may also “consume” coagulation factors locally leading to severe DIC. This is most likely the clinical scenario in our case. Understanding the role of coagulation factors in CHF is a developing area. Thus, early recognition of DIC is essential for implementation of appropriate management with anticoagulation to achieve better clinical outcomes.
